# Impact of Inhalation Sedation on Cardiovascular Haemodynamics in Hypertensive Patients Requiring Dental Treatment: A Clinical Observational Study

**DOI:** 10.3290/j.ohpd.c_2753

**Published:** 2026-06-29

**Authors:** Hassan Abed

**Affiliations:** a Hassan Abed Associate Professor, Department of Basic and Clinical Oral Sciences, College of Dental Medicine, Umm AL–Qura University, Makkah, Saudi Arabia. Study design, conducting the study, analysing the data, writing and reviewing the manuscript.

**Keywords:** dental anxiety, dental extraction, dental pain, hypertension, inhalation sedation

## Abstract

**Background:**

Several research studies reported the anxiolytic and analgesic properties of nitrous oxide sedation, as well as its effectiveness in facilitating dental procedures for anxious populations, specifically, dental patients with high blood pressure.

**Objective:**

This clinical observational study aims to assess the impact of inhalation sedation using nitrous oxide mixed with oxygen on cardiovascular haemodynamics in hypertensive patients requiring dental treatment.

**Methods and Materials:**

Adult dental patients with hypertension and dental anxiety who attended the dental clinic with acute dental pain were included in the study. Non-invasive blood pressure (NIBP/mmHg), heart rate (beats per min (bpm)), and oxygen saturation (SPO2%) were measured before, during, and after inhalation sedation. Participants’ dental anxiety and need for conscious sedation were measured using the Modified Dental Anxiety Scale and the Indicator of Sedation Need, respectively. Dental procedure characteristics and sedation details were also collected, such as procedure type and duration, number of visits, and nitrous oxide concentration.

**Results:**

Significant reductions in heart rate and blood pressure were observed during and after the procedure compared with baseline. Mean heart rate decreased from pre-sedation to intraprocedural and post-procedural phases, while blood pressure shifted from pre-hypertensive or hypertensive levels to normal in most participants during and after sedation. Respiratory rate remained stable, and oxygen saturation was maintained at optimal levels throughout all phases.

**Conclusion:**

Inhalation sedation with nitrous oxide and oxygen was associated with reductions in heart rate and blood pressure in hypertensive patients undergoing dental treatment, while maintaining stable oxygen saturation. It was also associated with reduced dental anxiety and facilitation of dental procedures. These findings suggest a potentially beneficial role for inhalation sedation in this population; however, causal inferences are limited by the observational study design.

Dental anxiety is considered a major barrier to seeking dental care.^34^ Poor oral health status related to dental anxiety negatively impacts oral health status and oral health-related quality of life.^24^ People with dental anxiety usually attend a dental clinic with complex dental needs due to the delay in seeking dental treatment and the low possibility of having dental treatment by other pharmacological techniques, such as conscious sedation or general anaesthesia, which both require training and arrangement.^24^

Conscious sedation techniques such as inhalation, intravenous, intranasal, or oral sedation have become necessary in delivering dental care for many patients with dental anxiety. These techniques provide a positive patient experience and reduce the patient’s negative thoughts toward dentistry.^24^

Consider using conscious sedation techniques, specifically inhalation sedation using nitrous oxide mixed with oxygen, which has a meaningful impact when delivering dental treatment for dental patients with high blood pressure.^6^ Several research studies reported the anxiolytic and analgesic properties of nitrous oxide sedation, as well as its effectiveness in facilitating dental procedures for anxious populations.^2,13,14,27,28,31^ Studies showed the positive vagolytic cardiovascular effects of inhalation anaesthetics and the stabilising impact of nitrous oxide during dental procedures.^5,13,17,29,30^ Besides, inhalation sedation induces a dose-dependent stabilisation of cardiovascular parameters.^11,19,26,30,35^

There are gaps in the literature regarding the haemodynamic effects of inhalation sedation using nitrous oxide mixed with oxygen in patients diagnosed with hypertension. Hypertensive individuals are more susceptible to stress-related cardiovascular fluctuations during dental procedures. Despite findings of the previously published studies on the anxiolytic and general physiological effects of inhalation sedation in fit and well dental patients, evidence specifically addressing hypertensive patients in routine clinical dental settings remains limited. Therefore, assessing the haemodynamic responses to inhalation sedation in hypertensive patients within an observational clinical framework is crucial to understanding its potential role and safety profile in these higher-risk dental patients. Accordingly, this clinical observational study aims to assess the impact of inhalation sedation using nitrous oxide mixed with oxygen on cardiovascular haemodynamics in hypertensive patients requiring dental treatment.

## METHODS AND MATERIALS

### Study Setting and Measures

This was a clinical observational study and was conducted from September 2023 to October 2025. Dental adult patients with hypertension and dental anxiety who attended the dental clinic at Private Dental Clinics (Jeddah, Saudi Arabia) with acute dental pain were included in the study. Hypertension status was determined based on diagnosed cases by physicians that were documented in the patient’s clinical records and/or current use of antihypertensive medication. Validated and reliable tools were used to measure the study’s outcomes. For example, demographic details such as age, gender, American Society of Anesthesiologists (ASA) classifications,^12^ medical history, mental capacity based on the Mental Capacity Act,^22^ and socioeconomic status were collected from the patient’s medical file. Non-invasive blood pressure (NIBP/mmHg), heart rate (beats per min (bpm)), and oxygen saturation (SPO_2_%) were measured before, during, and after inhalation sedation using an automated portable machine (PHILIPS, SureSigns VS4). Participants’ dental anxiety and need for conscious sedation were measured using the Modified Dental Anxiety Scale (MDAS),^16^ and the Indicator of Sedation Need (IOSN),^34^ respectively. Dental procedure characteristics and sedation details were also collected, such as procedure type and duration, number of visits, and nitrous oxide concentration (N_2_O%).

### Statistical Analyses

All statistical analyses were performed using IBM SPSS Statistics for Windows, Version 27.0 (IBM, Armonk, NY, USA). Data were initially assessed for completeness and normality. Descriptive statistics were generated for demographic and clinical characteristics, including frequencies and percentages for categorical variables, and means with standard deviations (SD) for continuous variables. For haemodynamic parameters (ie, heart rate, NIBP, and oxygen saturation), repeated measures at three time points before sedation, during the procedure, and after the procedure were analysed using repeated-measures ANOVA for normally distributed data, with Bonferroni-adjusted pairwise comparisons to identify differences between specific time points. When normality assumptions were violated, the Friedman test with post-hoc Wilcoxon signed-rank tests and Bonferroni correction was applied. Associations between patient characteristics (ie, age, sex, ASA classification, medical history, mental capacity, socioeconomic status) and baseline heart rate were examined using multiple linear regression analysis, with pre-sedation heart rate as the dependent variable. Variables with *P* < 0.20 in univariate analysis were entered into the multivariable model. A two-tailed *P* value < 0.05 was considered statistically significant for all analyses.

## RESULTS

### Participant Characteristics

A total of 60 hypertensive patients presenting with acute dental pain were included in the analysis. The demographic and clinical characteristics are shown in Table 1. The mean age was 33.1 ± 17.1 years (range: 11–89), with a slightly higher proportion of females than males. Most patients were classified as ASA I, with smaller proportions classified as ASA II or III. The majority had no significant comorbidities aside from hypertension, and mental capacity was preserved in most participants. Nearly half belonged to the moderate socioeconomic category.

**Table 1:** Baseline demographic and clinical characteristics of participants (N = 60)

Variable	n	%	Mean ± SD / range
**Age (years)**	60		33.1 ± 17.1 (11–89)
< 20 years old	11	18.3	
20 ≠ 29 years old	18	30.1	
30–39 years old	20	33.3	
40 years old and above	11	18.3	
**Gender**			
Male	28	46.7	
Female	32	53.3	
**ASA classification**	60		
ASA I	52	86.7	
ASA II	2	3.3	
ASA III	6	10.0	
**Medical history**			
With medical history	18	30.0	
Fit and well	42	70.0	
**Mental capacity**			
Have mental capacity	50	83.3	
Lacking of mental capacity	10	16.7	
**Socioeconomic status**			
Low	7	11.7	
Moderate	29	48.3	
High	24	40.0	
Note: Values are presented as n (%), unless otherwise stated. ASA = American Society of Anesthesiologists.			

### Dental Procedure and Sedation Characteristics

The majority of procedures were dental extractions, followed by restorative treatments and dental scaling. The mean duration of procedures was approximately 49 min, with a mean nitrous oxide concentration of 50%. Pre-sedation anxiety levels were high, with most patients classified as having high or very high anxiety on the MDAS. Despite this, pain scores during treatment were low, and both patient and dentist treatment success ratings were high (Table 2).

**Table 2:** Dental procedure characteristics and sedation details (N = 60)

Variable	n	%	Mean ± SD / range
**Procedure type**	60		
Dental extraction	52	86.7	
Dental implant	1	1.7	
Dental restoration	3	5.0	
Scaling	3	5.0	
Sinus lifting	1	1.7	
**Duration (min)**	60		49.0 ± 16.9 (15–85)
**Number of visits**	60		1.5 ± 1.4 (0–7)
**Nitrous oxide concentration (%)**	60		50.0 ± 13.4 (0–7)
**Pre-sedation MDAS**			
Moderate dental anxiety	5	8.3	
High dental anxiety	18	30.0	
Very high dental anxiety	37	61.7	
**Pain score (VAS)**	60		0.9 ± 1.5 (0–5)
**Treatment success as reported by dentists**			
Totally unsuccessful	1	1.7	
Neutral	1	1.7	
Successful	7	11.7	
Very successful	51	85.0	
**Treatment success as reported by patients**			
Totally unsuccessful	1	1.7	
Neutral	5	8.3	
Successful	10	16.7	
Very successful	44	73.3	


### Haemodynamic Parameters

Significant reductions in heart rate and NIBP were observed during and after the procedure compared with baseline. Mean heart rate decreased from 97.4 ± 15.3 pre-sedation to 84.2 ± 9.6 bpm intraprocedurally and to 81.9 ± 9.3 bpm post-procedurally. Therefore, there was an overall reduction of approximately 15.5 bpm.

At the same time, NIBP shifted from pre-hypertensive or hypertensive levels (almost 40% of the participants) to normal during (8.3%) and after sedation (no cases). Respiratory rate remained stable, and oxygen saturation was maintained at optimal levels throughout all phases (Table 3).

**Table 3:** Haemodynamic parameters before, during, and after inhalation sedation

Parameter	Before	During	After
**Heart rate (bpm)**	97.4 ± 15.3 (68–129)	84.2 ± 9.6 (55–101)	81.9 ± 9.3 (65–100)
**NIBP (mmHg)**			
Normal	36 (60.0)	55 (91.7)	60 (100.0)
Pre-hypertension	19 (31.7)	5 (8.3)	0 (0.0)
Hypertension 1	5 (8.3)	0 (0.0)	0 (0.0)


### Heart Rate Comparisons

Pairwise comparisons showed statistically significant differences in heart rate between all time points. Heart rate significantly decreased by a mean of 13.2 bpm from before to during sedation, and by 15.5 bpm from before to after the procedure, with a smaller but significant (almost 2.3 bpm) decrease between during and after (all *P* < 0.001) – see Table 4. Similarly, for NIBP, a repeated-measures analysis was conducted to examine changes across the three time points. Results are presented in Table 5, which shows a significant time effect, indicating that NIBP decreased during sedation and remained lower post-procedure compared with baseline values.

**Table 4:** Pairwise comparisons of heart rate across time points (Bonferroni adjusted)

Comparison	Mean difference	SE	95% CI	P value
Before vs during	13.22	1.27	10.10–16.33	< 0.001
Before vs after	15.50	1.32	12.25–18.75	< 0.001
During vs after	2.28	0.32	1.49–3.08	< 0.001


**Table 5:** Tests of within-subject effects for non-invasive blood pressure across three time points

Source	Type III sum of squares	df	Mean square	F	P value	Partial eta squared
Time (before–during–after)	254.422	1	254.422	1053.808	< 0.001	0.947
Error (time)	14.244	59	0.241			
Repeated-measures ANOVA was used to assess changes in NIBP across the three time points. Bonferroni correction applied for pairwise comparisons.						

### Factors Associated with Haemodynamic Changes

Multivariate regression identified mental capacity and socioeconomic status as significant predictors of pre-sedation heart rate. Patients with preserved mental capacity had higher pre-sedation heart rates, while those in the moderate socioeconomic group exhibited lower rates compared to the high socioeconomic group. No other variables, including age, gender, ASA classification, medical history, or medication use, were significantly associated with heart rate at any time point (Table 6). The corresponding multivariable analysis for NIBP is presented in Table 7. While fewer predictors reached statistical significance compared to heart rate, socioeconomic status showed a notable influence on baseline NIBP, with no strong associations found for intra- and post-procedural values after adjustment.

**Table 6:** Factors associated with heart rate before, during, and after procedures using multiple regression models

Dependent variable: heart rate		B	SE	95% CI	P value
**Before**	Intercept	81.72	13.48	54.62–108.82	< 0.001
	Age = < 20 years old	–5.24	7.35	–20.01–9.54	0.480
	Age = 20–29 years old	–0.68	5.86	–12.47–11.11	0.908
	Age = 30–39 years old	–4.31	5.70	–15.78–7.15	0.453
	Gender = Male	–4.57	3.73	–12.06–2.92	0.226
	Medical history = with medical history	2.87	6.32	–9.83–15.57	0.652
	Mental capacity = have mental capacity	25.10	9.45	6.11–44.09	0.011a
	ASA = ASA I	2.01	15.97	–30.10–34.11	0.901
	ASA = ASA II	–19.48	15.08	–49.79–10.83	0.202
	Medication = with medication	9.30	12.62	–16.08–34.67	0.465
	Socioeconomic status = low	2.44	7.30	–12.23–17.11	0.74
	Socioeconomic status = moderate	–8.87	3.94	–16.79–0.95	0.029
**During**	Intercept	75.78	8.80	58.10–93.47	< 0.001
	Age = < 20 years old	–5.00	4.80	–14.64–4.64	0.302
	Age = 20–29 years old	–0.68	3.83	–8.38–7.01	0.859
	Age = 30–39 years old	–1.28	3.72	–8.76–6.20	0.732
	Gender = Male	–1.19	2.43	–6.08–3.70	0.627
	Medical history = with medical history	–1.33	4.12	–9.61–6.97	0.749
	Mental capacity = have mental capacity	10.75	6.17	–1.65–23.15	0.088
	ASA = ASA I	3.01	10.42	–17.94–23.96	0.774
	ASA = ASA II	–0.60	9.84	–20.38–19.18	0.952
	Medication = with medication	2.68	8.24	–13.88–19.24	0.746
	Socioeconomic status = low	3.92	4.76	–5.65–13.50	0.414
	Socioeconomic status = moderate	–3.20	2.57	–8.37–1.96	0.219
**After**	Intercept	73.48	8.76	55.86–91.09	< 0.001
	Age = < 20 years old	–5.40	4.78	–15.00–4.21	0.264
	Age = 20–29 years old	–1.29	3.81	–8.95–6.37	0.736
	Age = 30–39 years old	–2.77	3.71	–10.22–4.68	0.459
	Gender = Male	–1.12	2.42	–5.99–3.75	0.645
	Medical history = with medical history	–1.59	4.11	–9.85–6.67	0.700
	Mental capacity = have mental capacity	9.94	6.14	–2.41–22.29	0.112
	ASA = ASA I	3.44	10.38	–17.43–24.30	0.742
	ASA = ASA II	–3.79	9.80	–23.49–15.92	0.701
	Medication = with medication	4.15	8.20	–12.35–20.64	0.615
	Socioeconomic status = low	3.82	4.74	–5.72–13.36	0.424
	Socioeconomic status = moderate	–1.14	2.56	–6.28–4.01	0.659


**Table 7:** Multivariable regression analysis for predictors of non-invasive blood pressure across time points

Variable	Baseline NIBP β (95% CI)	P value	Intraprocedural NIBP β (95% CI)	P value	Post-procedural NIBP β (95% CI)	P value
**Age (years)**	0.00 (–0.01–0.01)	0.557	0.00 (–0.01–0.00)	0.595	–2.2E–17 (–2.2E–17–2.2E–17)	–
**Sex (male vs. female)**	0.11 (–0.21–0.42)	0.492	0.10 (–0.04–0.24)	0.164	6.0E–17 (6.0E–17–6.0E–17)	–
**ASA classification (I vs. II/III)**	–0.47 (–1.76–0.81)	0.463	–0.33 (–0.91–0.24)	0.25	–1.0E–15 (–1.0E–15–1.0E–15)	–
**Mental capacity (preserved vs not)**	0.33 (–0.30–0.97)	0.296	0.06 (–0.23–0.34)	0.685	3.5E–16 (3.5E–16–3.5E–16)	–
**Socioeconomic status (high vs moderate)**	0.11 (–0.22–0.45)	0.507	–0.07 (–0.22–0.08)	0.341	1.4E–16 (1.4E–16–1.4E–16)	–
**Medical history (hypertension only)**	–0.11 (–1.29–1.08)	0.859	0.06 (–0.47–0.59)	0.808	–4.7E–16 (–4.7E–16–4.7E–16)	–
**Medications (yes vs no)**	0.63 (–0.23–1.49)	0.147	0.03 (–0.35–0.42)	0.86	3.5E–17 (3.5E–17–3.5E–17)	–
**Constant**	1.28 (0.10–2.46)	0.034	1.36 (0.83–1.89)	< 0.001	1.0E+00 (1.0E+00–1.0E+00)	–
Linear regression models with NIBP (mmHg) as the dependent variable. Variables with *P* < 0.20 in univariate analysis entered into the multivariable model. CI: Confidence interval.						

### Adverse Events

Adverse events were generally mild and transient (Table 8). The most common was a light headache, followed by chills and crying. Isolated cases of bradycardia and tachycardia occurred, but no severe complications or oxygen desaturation events were recorded. All patients recovered uneventfully without escalation of care.

**Table 8:** Reported adverse events and side effects during and after sedation

Adverse event	n	%
Light headache	5	31.3
Chills	2	12.5
Crying	2	12.5
Bradycardia	1	6.2
Tachycardia	1	6.2
Other	5	31.3


## DISCUSSION

This study evaluated the impact of inhalation sedation on cardiovascular haemodynamics in hypertensive patients presenting with acute dental pain, while also assessing its anxiolytic effects and treatment outcomes. Significant reductions in heart rate and NIBP were observed during and after sedation compared with baseline. Oxygen saturation remained stable, and adverse events were mild and transient. Inhalation sedation facilitated successful dental procedures in patients with high baseline anxiety, supporting its role as both a safe and effective behavioural and physiological management tool.^7,13,27,28^

The haemodynamic changes observed are consistent with established evidence that nitrous oxide exerts dose-dependent vagolytic and anxiolytic effects, resulting in decreased heart rate and blood pressure without compromising oxygenation.^2,27,18,25^ Prior physiological studies have demonstrated that nitrous oxide attenuates sympathetic outflow while enhancing vagal tone, leading to cardiovascular stabilisation during stressful stimuli such as dental extractions.^1,8,26,30,35^ Similar findings have been documented in paediatric and adult dental settings, where inhalation sedation reduces perioperative cardiovascular variability and improves patient tolerance.^10,13,27^

The observed reduction in dental anxiety is consistent with systematic reviews and clinical trials showing that nitrous oxide sedation effectively lowers MDAS scores and behavioural distress during dental procedures.^14,27,28,35^ Its rapid onset, anxiolytic effect, and minimal recovery time make it a preferred first-line sedation technique in a primary dental setting.

Nitrous oxide’s cardiovascular effects are mediated through both central and peripheral mechanisms. It causes mild myocardial depression balanced by sympathetic activity in healthy individuals but predominantly induces vagolytic and anxiolytic effects in hypertensive or anxious patients, leading to reductions in heart rate and blood pressure.^3,8,11,15,23,30,32,35^

These responses are advantageous in dental extractions, which typically elicit transient sympathetic surges due to nociception and fear.^1,9,20^ Maintaining optimal oxygenation throughout the procedure further minimises hypoxic complications, a practice endorsed by current sedation guidelines.^1,2,10,13,33^

Clinically, these effects are significant. Hypertensive patients undergoing dental procedures are at increased risk of cardiovascular events triggered by procedural stress and pain. By stabilising haemodynamic responses and alleviating anxiety, inhalation sedation can reduce the need for general anaesthesia or systemic sedatives, which may carry higher cardiovascular risks.^4,21,23,33,36^ This aligns with contemporary guidelines promoting conscious sedation as the preferred approach for ASA I–II patients, including those with controlled hypertension.^1,2,4,10,21,33^

### Strengths and Limitations

A major strength of this study is the focus on a hypertensive patient population – a group for whom sedation choices must balance anxiolytic benefits with cardiovascular safety. The integration of anxiety and haemodynamic assessments provides a more holistic understanding of sedation effects in real-world dental practice. Furthermore, standardised nitrous oxide administration and continuous oxygen delivery ensured procedural consistency and safety.

However, several limitations should be noted. For example, there was no comparison group; therefore, it was not possible to observe an actual reduction in heart rate and blood pressure solely due to nitrous oxide and oxygen. The sample size was modest and drawn from a single centre, which may limit external generalisability. Haemodynamic monitoring relied on non-invasive methods; future studies incorporating heart rate variability or invasive arterial monitoring could offer deeper insights into autonomic modulation. Additionally, while MDAS is a validated tool, it may not capture all psychosocial dimensions of dental anxiety. Lastly, it is also crucial to mention that findings should be interpreted as associations rather than direct effects of the sedation intervention.

### Future Research Directions

Future studies should adopt multicenter designs with larger, stratified samples to validate these findings across different populations and dental procedures. Dose–response studies could clarify optimal nitrous oxide concentrations for haemodynamic control in hypertensive patients. Longitudinal studies examining repeated sedation exposures may also provide insights into adaptive anxiety responses and cardiovascular conditioning over time. Finally, integrating psychophysiological markers, such as cortisol levels or heart rate variability, could enhance understanding of the interplay between sedation, anxiety, and cardiovascular regulation.

## CONCLUSIONS

Inhalation sedation with nitrous oxide and oxygen helps reduce heart rate and blood pressure while maintaining stable oxygenation in hypertensive patients undergoing dental treatment. It also provides substantial anxiolysis that facilitates successful dental procedures. These effects highlight that inhalation sedation can be a useful behavioural and physiological management approach for hypertensive patients in dental settings.

### Ethical approval

The study was approved by the Institutional Review Board of (Number: HAPO-02-K-012-2025-09-2873).
